# Brian [18F]FDG PET associations of cervical cancer-related peripheral inflammatory markers

**DOI:** 10.3389/fonc.2025.1598911

**Published:** 2025-06-26

**Authors:** Yao Hu, Yuan Zhong, Yuxiao Hu

**Affiliations:** ^1^ Department of PET/CT Center, Jiangsu Cancer Hospital and Jiangsu Institute of Cancer Research and the Affiliated Cancer Hospital of Nanjing Medical University, Nanjing, China; ^2^ School of Psychology, Nanjing Normal University, Nanjing, Jiangsu, China; ^3^ Jiangsu Key Laboratory of Mental Health and Cognitive Science, School of Psychology, Nanjing Normal University, Nanjing, Jiangsu, China; ^4^ National Health Commission Key Laboratory of Nuclear Medicine and Jiangsu Key Laboratory of Molecular Nuclear Medicine, Wuxi, China

**Keywords:** regional brain glucose metabolism, cervical cancer, peripheral inflammatory markers, tumor metabolic parameters, PET

## Abstract

**Objective:**

The purpose of this study was to explore brain metabolic correlates of peripheral inflammatory markers in patients with cervical cancer (CC).

**Methods:**

Cervical cancer (CC) patients (267) without treatments who underwent [18F] Fluorodeoxyglucose ([18F]FDG) positron-emission tomography (PET)/computed tomography (CT) were retrospectively studied. All CC patients were grouped into the International Federation of Gynecology and Obstetrics (FIGO) stage II (n=16), the FIGO stage III (n=160), and the FIGO stage IV (n=91) according to the FIGO stage. According to the median of metabolic tumor volume (MTV) or total lesion glycolysis (TLG) for primary tumor in different FIGO stage, CC patients in different FIGO stage were grouped into the Low_MTV (TLG) group (<median) and High_MTV (TLG) group (≥median). Regression analysis were used to explore the relationships between regional brain glucose metabolism and peripheral inflammatory markers [including Neutrophil-to-Lymphocyte ratio (NLR), Platelet-to-Lymphocyte ratio (PLR), Monocyte-to-Lymphocyte ratio (MLR), and systemic immune-inflammation index (SII)] in whole group, subgroups in different FIGO stage.

**Results:**

The MTV and TLG for primary tumor positively correlated with SII (*r_MTV_* =0.402, *P_MTV_* =0.000; *r_TLG_* =0.397, *P_TLG_* =0.000), PLR (*r_MTV_* =0.317, *P_MTV_* =0.000; *r_TLG_* =0.323, *P_TLG_* =0.000), NLR (*r_MTV_* =0.311, *P_MTV_* =0.000; *r_TLG_* =0.328, *P_TLG_* =0.000), MLR (*r_MTV_* =0.255, *P_MTV_* =0.001; *r_TLG_* =0.275, *P_TLG_* =0.000) in FIGO stage III, and they positively correlated with SII (*r_MTV_* =0.223, *P_MTV_* =0.033; *r_TLG_* =0.291, *P_TLG_* =0.005), NLR (*r_TLG_* =0.220, *P_TLG_* =0.036) in FIGO stage IV, but didn’t significantly correlate with MLR, or PLR in FIGO stage IV or with all peripheral inflammatory markers in FIGO stage II (P>0.05). The SII and NLR had significantly negative correlations with the glucose metabolism mainly in the bilateral dorsolateral prefrontal cortex (dlPFC) in CC patients with FIGO stage III (*P_FWEc_* < 0.05) and those regions negatively correlated with SII were mainly located in the right dlPFC in CC patients with FIGO stage IV (*P_FWEc_* < 0.05). Compared with patients with the Low_MTV (TLG) group in FIGO stage III, those with High_MTV (TLG) group showed stronger relationships between the glucose metabolism of dlPFC and peripheral inflammatory markers (SII, or NLR), while the relationships in FIGO stage IV weakened or even disappeared.

**Conclusion:**

The glucose metabolism in the dlPFC negatively correlated with peripheral inflammatory markers in CC patients with FIGO stage III or IV may be relevant in the disease severity and vary depending on the disease severity.

## Introduction

The nervous system play a role in the cancer aetiopathogenesis. The communication between brain and tumor tissue is through the bid-rectional regulation of neural, humoral pathways and neuroendocrine-immune system (1). The previous neuroimaging studies by using [18F]FDG PET/CT have confirmed that cancer patients without treatment showed abnormal metabolism in some brain regions (2, 3)(insula, basal ganglia, thalamus, hippocampus, amygdala, prefrontal cortex, and cerebellum), which may indicate the underlying associated of tumor tissue and nervous system.

[18F]fluorodeoxyglucose ([18F]FDG) positron-emission tomography (PET)/computed tomography (CT), as a whole-body noninvasive imaging method, can not only provide information of primary tumor burden, but also obtain whole brain PET [18F]FDG data. The PET metabolic parameters of primary tumor tissue, such as metabolic tumor volume (MTV) and total lesion glycolysis (TLG), could reflect the local tumor burden which including local metabolism of tumor tissue itself and the local inflammatory. Peripheral inflammatory markers, including the neutrophil-to-lymphocyte ratio (NLR), platelet-to-lymphocyte ratio (PLR), monocyte-to-lymphocyte ratio (MLR), and systemic immune-inflammation indices (SII) have been identified closely associated with local inflammatory of primary tumor that are considered correlated with worse outcome of patients with many cancer types, including lung cancer (4), cervical cancer (CC) (5), and renal cell carcinoma (6) and so on. Moreover, a meta-analysis of functional neuroimaging studies revealed that the prefrontal and temporal cortices, anterior cingulate cortex, amygdala, thalamus, hippocampus, hypothalamus, basal ganglia (striatum and caudate), insula, and midbrain/brainstem associated with peripheral inflammation in humans (7). Drawing on the above studies, the peripheral inflammatory markers may play critical roles in abnormalities of regional brain metabolism associated with tumor tissue.

However, the regional brain metabolism involved in the brain’s response to peripheral inflammatory markers and primary tumor burden have not been well characterized in CC patients. The purpose of this study was to use brain [18F]FDG PET/CT to explore the neural associations of CC-related peripheral inflammatory markers. We hypothesized that the peripheral inflammatory markers in CC patients would be associated with altered brain glucose metabolism and that relationships would be stronger for patients with the higher level of tumor burden.

## Experimental procedures

### Subjects

All CC patients who conducted brain [18F]FDG PET/CT scanning after whole-body PET/CT examination from May 2016 to May 2023 were retrospectively studied. The inclusion criteria were as follows: (a) squamous cell carcinoma of the cervix; (b) 18 ≤ age < 80; (c) without any treatments; and (d) right-handed subjects. The exclusion criteria were as follows: (a) CC patients with emotional, psychiatric, or neurological disorders; (b) with brain metastasis, trauma, and brain radiation; (c) with other cancers; (d) with serious liver and kidney dysfunction, pregnant, or breastfeeding; (e) images with defects or artifacts. Clinical data such as age, weight, height, [18F]FDG (mCi, injected dose), the FIGO stage and peripheral inflammatory markers (e.g., Neutrophil, Lymphocyte, Monocyte, and Platelet counts) from blood routine examination closest to the day of PET/CT scanning were collected. NLR, PLR, MLR, and SII were calculated using the formula Neutrophil counts/Lymphocyte counts, Platelet counts/Lymphocyte counts, Monocyte counts/Lymphocyte counts, and Platelet counts×Neutrophil counts/Lymphocyte counts, respectively.

### 18F-FDG PET/CT scanning

All patients were acquired above six hours of fasting and their blood glucose levels were less than or equal to 11.1 mmol/L before injection of [18F]FDG (3.7-7.4 MBq/kg). All patients with their eyes closed rest in a warm, quiet, and dark room about 50–70 min after injection. Patients underwent by the whole-body [18F]FDG PET/CT (Discovery 710, General Electric Medical Systems, Milwaukee, WI, USA). After whole-body PET/CT examination, a brain PET/CT scanning with one bed position (5 min/bed position) was performed by a 3D-mode standard technique (192 × 192 matrix) and a head non-contrast CT scan (300 mA; 120 kV) was used for anatomic localization and attenuation correction. The [18F]FDG PET data were reconstructed with using ordered subsets expectation maximization algorithm, 24 subsets, 2 iterations.

### [18F]FDG PET images preprocessing

The whole-body [18F]FDG PET images in DICOM format were transferred to the Beth Israel PET/CT viewer plugin for FIJI. The MTV and TLG for primary lesion of CC patients were delineated semiautomatically by FIJI (http://sourceforge.net/projects/bifijiplugins/). The auto segmentation algorithm identifies focal uptakes in the image and then from these uptakes a region-growing algorithm defines the region of interest (SUV threshold based on 41% of the SUVmax) around the tumor. The brain [18F]FDG PET images of CC patients were analyzed by use of statistical parametric mapping (SPM12, Wellcome Department of Cognitive Neurology, London, UK) implemented in MATLAB 2013b (Mathworks, Natick, Massachusetts, USA). The PET images in DICOM format were converted to images in NifTI format for following processing. The brain [18F]FDG images in NifTI format were spatially normalized into the Montreal Neurological Institute space (MNI) (bounding box: -90 -126 -72; 90 90 108, voxel size: 2 × 2 × 2 mm^3^). The spatially normalized brain [18F]FDG images were smoothed by use of an isotropic Gaussian filter with a 10-mm full width at one-half maximum.

### Statistical analysis

The [18F]FDG PET metabolic parameters of primary tumor and peripheral inflammatory markers of CC patients were analyzed by use of SPSS 25.0 software (SPSS, IL, USA), and *P* < 0.05 was considered significant. Data were expressed as median (range). The correlations between the [18F]FDG PET metabolic parameters of primary tumor and peripheral inflammatory markers were evaluated by use of the Spearman’s correlation. Peripheral inflammatory markers between different FIGO stages were analyzed by Kruskal-Wallis H Test. Firstly, according to FIGO stage, 267 CC patients were grouped into the FIGO stage II (n=16), the FIGO stage III (n=160), and the FIGO stage IV (n=91). Then, according to the median of MTV or TLG in different FIGO stage, 16 CC patients with FIGO stage II were grouped into the Low_MTV group (<13.28, n=8) and High_MTV group (≥13.28, n=8), or the Low_TLG group (<111.07, n=8) and High_TLG group (≥111.07, n=8), 160 CC patients with FIGO stage III were grouped into the Low_MTV group (<30.28, n=80) and High_MTV group (≥30.28, n=80), or the Low_TLG group (<287.97, n=80) and High_TLG group (≥287.97, n=80), 91 patients with FIGO stage IV were grouped into the Low_MTV group (<36.51, n=45) and High_MTV group (≥36.51, n=46), or the Low_TLG group (<341.34, n=45) and High_TLG group (≥341.34, n=46). Statistical differences between groups [Low_MTV (TLG) and High_MTV (TLG) groups] were evaluated by t-test or Mann-Whitney U test. Mann-Whitney U test was performed for data in accordance with non-normal distribution, while normal distribution data were analyzed by t-test. The correlations between the brain glucose metabolism from the whole CC patients or different subgroups and peripheral inflammatory markers were calculated by use of regression analysis with age as a covariate. A threshold of *P* < 0.05 family-wise error correction (FWE) at cluster level and with a threshold of *P <* 0.001 (uncorrected) was set in this study. And the voxels of minimum cluster was greater than 100. The results of the SPM r maps were analyzed with MRIcroGL and Xjview to show and found the corresponding anatomical locations of significant metabolic clusters. Results were displayed with brain regions (AAL), voxels, R score (correlation coefficient), or the MNI coordinates (x, y, z) of the peak voxels.

## Results

### Clinical characteristics

A total of 267 CC patients were included in this study. Of these 267 CC patients, the median of age was 53 years (from 26 years to 76 years) (**Table 1**). Median and range of MTV and TLG of primary lesion and neutrophil counts, lymphocyte counts, monocyte counts, platelet counts, SII, PLR, NLR, and MLR and FIGO stage were summarized in **Table 1**. There were significant differences in SII (*H*=14.333, *P*=0.001), PLR (*H*=12.952, *P*=0.002), NLR (*H*=17.321, *P*=0.000), MLR (*H*=16.544, *P*=0.000), MTV (*H*=14.314, *P*=0.001), TLG (*H*=13.750, *P*=0.001) among different FIGO stage. SII (*P_II-III_* =0.002, *P_II-IV_* =0.000), PLR (*P_II-III_* =0.003, *P_II-IV_* =0.001), NLR (*P_II-III_* =0.011, *P_II-IV_* =0.000), MLR (*P_II-III_* =0.004, *P_II-IV_* =0.000), MTV (*P_II-III_* =0.013, *P_II-IV_* =0.001), and TLG (*P_II-III_* =0.003, *P_II-IV_* =0.001) in FIGO stage III or IV were significant higher than those in FIGO stage II, while there were not significant differences in SII (*P_III-IV_* =0.860), PLR (*P_III-IV_* =1.000), NLR (*P_III-IV_* =0.050), MLR (*P_III-IV_* =0.201), MTV (*P_III-IV_* =0.162), or TLG (*P_III-IV_* =0.880) between FIGO stage III and FIGO stage IV (**Supplementary Table S1**). There were no statistically significant differences in age (*P*>0.05), BMI (*P*>0.05), or [18F]FDG (*P*>0.05) between the Low_MTV (TLG) group and High_MTV (TLG) group in different FIGO stage (**Supplementary Table S2**). SII, PLR, NLR, and MLR were higher in the High_MTV (TLG) group than those in the Low_MTV (TLG) group from FIGO stage III (*P*<0.05) and so was SII in FIGO stage IV(*P*<0.05) (**Supplementary Table S2**). There were no significant differences in SII, PLR, NLR, or MLR between the Low_MTV (TLG) group and High_MTV (TLG) group in FIGO stage II (*P*>0.05) and neither were in PLR, NLR, or MLR in FIGO stage IV (*P*>0.05).

**Table 1:** Characteristics of patients with cervical cancer.

Characteristics	Patients (n=267)
Age (y, median, range)	53.0 (26, 76)
BMI (median, range)	23.44 (16.20, 45.17)
[18F]FDG (mCi) (median, range)	10.11 (5.98, 13.17)
MTV(cm^3^) (Median, Range)	31.03 (2.22-225.15)
TLG (g) (Median, Range)	292.00 (9.41, 2978.09)
Neutrophil counts (×10^9^/L, Median, Range)	4.28 (0.86, 14.47)
Lymphocyte counts (×10^9^/L, Median, Range)	1.53 (0.42, 3.84)
Monocyte counts (×10^9^/L, Median, Range)	0.42 (0.06, 1.20)
Platelet counts (×10^9^/L, Median, Range)	259.00 (66.0, 540.00)
SII (Median, Range)	698.80 (90.04, 5295.48)
PLR (Median, Range)	167.51 (61.72, 438.18)
NLR (Median, Range)	2.73 (0.65, 16.72)
MLR (Median, Range)	0.26 (0.08, 0.90)
FIGO Stage	
I [n (%)]	0 (0)
II [n (%)]	16 (6.0)
IV [n (%)]	160 (59.9)
IV [n (%)]	91 (34.1)

BMI, Body Mass Index; [18F]FDG, [18F]fluorodeoxyglucose; MTV, metabolic tumor volume; TLG, total lesion glycolysis; SII, Systemic immune-inflammation index; PLR, Platelet-to-Lymphocyte ratio; NLR, Neutrophil-to-Lymphocyte ratio; MLR, Monocyte-to-Lymphocyte ratio; FIGO, International Federation of Gynecology and Obstetrics.

### PET metabolic parameters of primary lesion correlated with peripheral inflammatory markers in different FIGO stage

The MTV and TLG for primary tumor positively correlated with SII (*r_MTV_* =0.402, *P_MTV_* =0.000; *r_TLG_* =0.397, *P_TLG_* =0.000), PLR (*r_MTV_* =0.317, *P_MTV_* =0.000; *r_TLG_* =0.323, *P_TLG_* =0.000), NLR (*r_MTV_* =0.311, *P_MTV_* =0.000; *r_TLG_* =0.328, *P_TLG_* =0.000), MLR (*r_MTV_* =0.255, *P_MTV_* =0.001; *r_TLG_* =0.275, *P_TLG_* =0.000) in FIGO stage III (**Table 2**). The MTV (or TLG) positively correlated with SII (*r_MTV_* =0.223, *P_MTV_* =0.033; *r_TLG_* =0.291, *P_TLG_* =0.005), NLR (*r_TLG_* =0.220, *P_TLG_* =0.036) in FIGO stage IV, but the MTV and TLG didn’t correlate with MLR, or PLR (P>0.05) and MTV didn’t correlated with NLR (*r_MTV_* =0.153, *P_MTV_* =0.148) (**Table 2**). There were no significant correlations between MTV (TLG) for primary tumor and SII, NLR, PLR, or MLR in FIGO stage II (P>0.05) (**Table 2**).

**Table 2:** PET metabolic parameters of primary lesion correlated with peripheral inflammatory markers in different FIGO stage.

Peripheral Inflammatory Markers	FIGO stage II				FIGO stage III				FIGO stage IV			
	MTV		TLG		MTV		TLG		MTV		TLG	
	*r*	*P*	*r*	*P*	*r*	*P*	*r*	*P*	*r*	*P*	*r*	*P*
SII	-0.012	0.965	0.018	0.948	0.402	0.000	0.397	0.000	0.223	0.033	0.291	0.005
PLR	0.165	0.542	0.091	0.737	0.317	0.000	0.323	0.000	0.103	0.330	0.133	0.209
NLR	0.224	0.405	0.097	0.721	0.311	0.000	0.328	0.000	0.153	0.148	0.220	0.036
MLR	0.134	0.621	0.050	0.854	0.255	0.001	0.275	0.000	0.087	0.413	0.133	0.209

### Correlation of peripheral inflammatory markers with brain glucose metabolism in different FIGO stage

**Figure 1:**
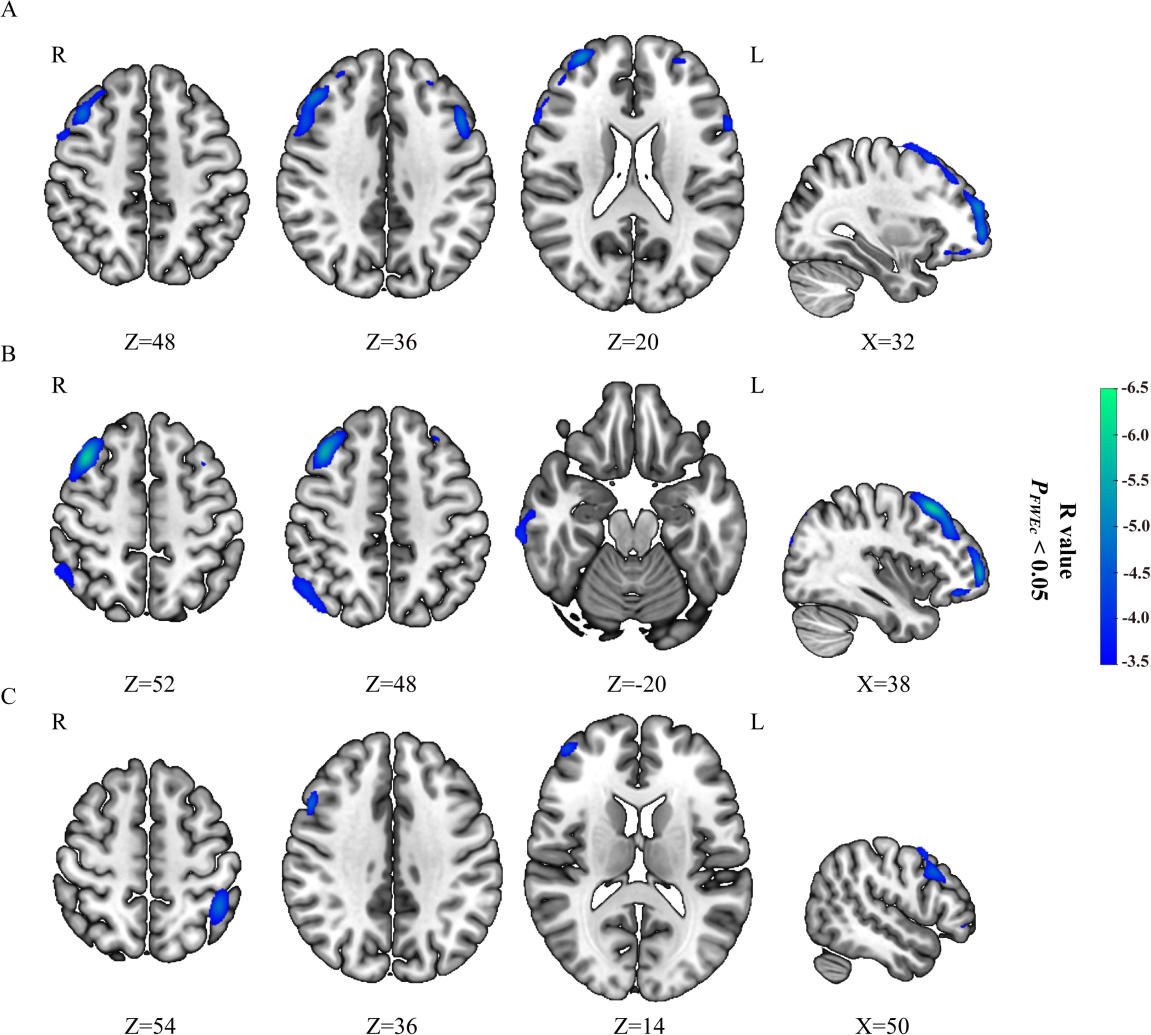
Correlation of peripheral inflammatory markers with brain glucose metabolism in FIGO stage III and IV. SII **(A)** and NLR **(B)** negatively correlated with brain glucose metabolism in patients with cervical cancer from FIGO stage III, while no significantly positive correlation were observed. SII **(C)** negatively correlated with brain glucose metabolism in patients with cervical cancer from FIGO stage IV, while no significantly positive correlation were observed. Hypometabolic regions correlated with peripheral inflammatory markers were shown in blue to green.

The SII and NLR had significantly negative correlations with the glucose metabolism mainly in the bilateral dorsolateral prefrontal cortex (dlPFC) in CC patients with FIGO stage III (*P_FWEc_* < 0.05) (**Table 3**, **Figures 1A, B**), but no significant correlations was observed between PLR (or MLR) and cerebral glucose metabolism. The brain regions negatively correlated with SII were mainly located in the right dlPFC in CC patients with FIGO stage IV (*P_FWEc_* < 0.05) (**Table 3**, **Figure 1C**), while the brain regions correlated with NLR were not observed. In addition, the other metabolic regions negatively correlated with SII and NLR in FIGO stage III were located in the Temporal_Mid_R, Temporal_Inf_R, Angular_R, and Parietal_Inf_R (*P_FWEc_* < 0.05), while the metabolic region correlated with SII in FIGO stage IV were located in Parietal_Inf_L (*P_FWEc_* < 0.05). SII, NLR, PLR, or MLR not significantly correlated with any brain metabolic regions in FIGO stage II.

**Table 3:** Peripheral inflammatory markers correlated with brain glucose metabolism in patients with cervical cancer in FIGO stage III or IV.

Peripheral Inflammatory Markers	Brain Regions (aal)	R Score	Voxels	Peak MNI Coordinates (x, y, z)
FIGO stage III				
SII				
	Frontal_Mid_R(L), Frontal_Sup_R(L), Frontal_Inf_Oper_R, Frontal_Inf_Tri_R	-5.17	5420	32, 54, 20
	Frontal_Inf_Tri_L, Frontal_Mid_L	-4.80	713	-46, 16, 36
	Temporal_Inf_R, Temporal_Mid_R	-4.66	569	60, -12, -38
	Angular_R, Parietal_Inf_R	-4.21	516	42, -72, 48
NLR				
	Frontal_Mid_R(L), Frontal_Inf_Tri_R(L), Frontal_Sup_R(L),Frontal_Sup_Medial_R, Frontal_Inf_Oper_R	-6.07	9399	38, 20, 52
	Angular_R, Parietal_Inf_R	-6.22	1060	50, -60, 48
	Temporal_Mid_R, Temporal_Inf_R	-4.09	511	68, -28, -20
PLR				
No suprathershold clusters				
MLR				
No suprathershold clusters				
FIGO stage IV				
SII				
	Frontal_Mid_R, Frontal_Inf_Oper_R, Frontal_Sup_R	-4.69	681	50, 16, 36
	Frontal_Mid_R, Frontal_Inf_Tri_R	-4.39	567	44, 50, 14
	Parietal_Inf_L	-4.87	513	-42, -46, 54
NLR				
No suprathershold clusters				

R Score, correlation coefficient; MNI, Montreal Neurological Institute space.

### Correlation of peripheral inflammatory markers with brain glucose metabolism in Low_MTV (TLG) group and High_MTV (TLG) group from FIGO stage III or IV

**Figure 2:**
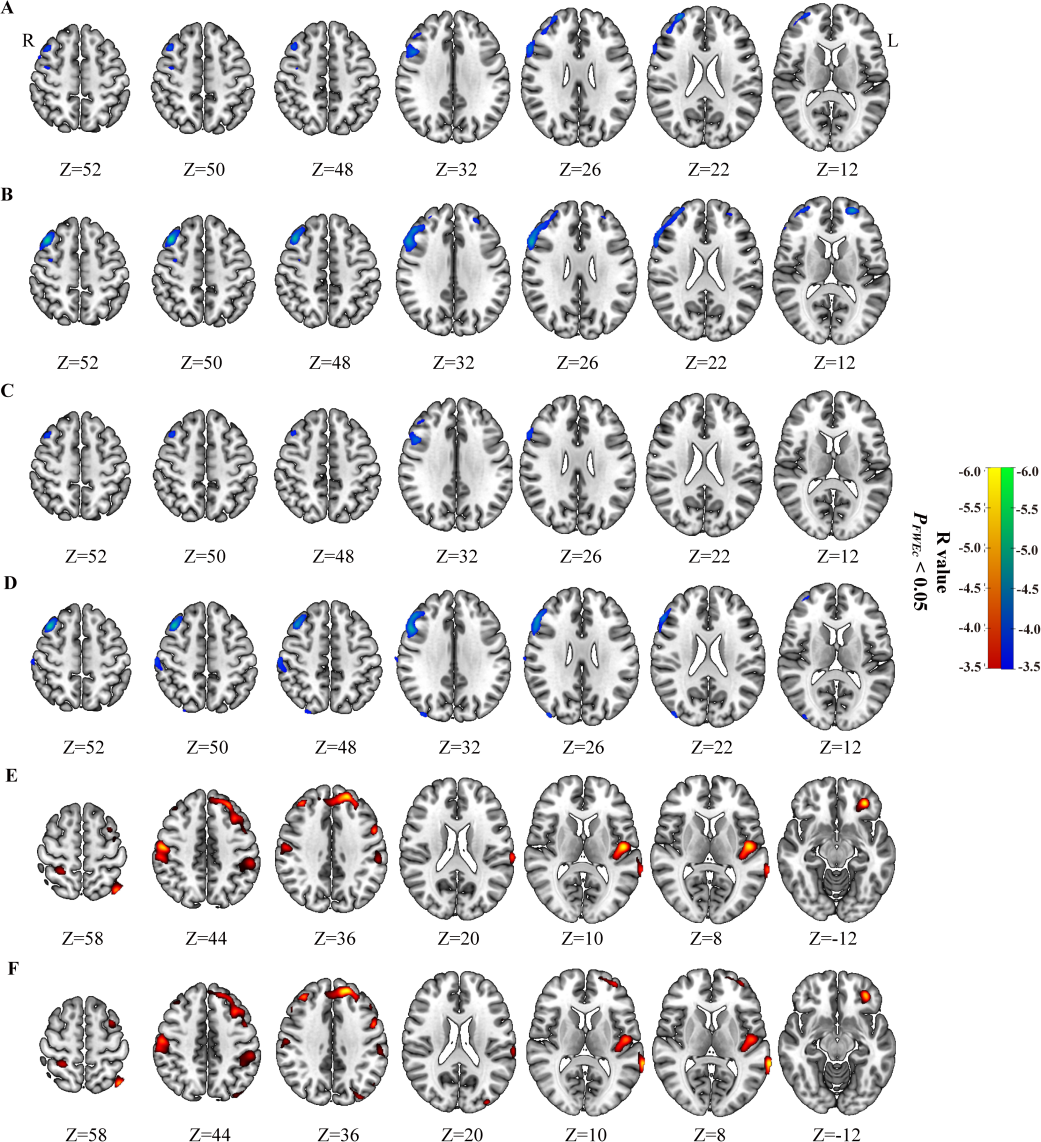
Correlation of peripheral inflammatory markers with brain glucose metabolism in Low_MTV (TLG) group and High_MTV (TLG) group from FIGO stage III and IV. SII **(A)** and NLR **(B)** negatively correlated with brain glucose metabolism in patients with cervical cancer in High_MTV group from FIGO stage III, but not found in Low_MTV group from FIGO stage III. SII **(C)** and NLR **(D)** negatively correlated with brain glucose metabolism in patients with cervical cancer in High_TLG group from FIGO stage III, but not found in Low_TLG group from FIGO stage III. SII negatively correlated with brain glucose metabolism in patients with cervical cancer in Low_MTV group **(E)** and Low_TLG group **(F)** from FIGO stage IV, but not found in High_MTV(TLG) group from FIGO stage IV. Hypometabolic regions correlated with peripheral inflammatory markers for Low_MTV (TLG) group were shown in red to yellow, and hypometabolic regions for High_MTV (TLG) group in blue to green.

The SII and NLR had negative correlations with the glucose metabolism in high_MTV(TLG) group from FIGO stage III (*P_FWEc_* < 0.05), but not found in Low_MTV(TLG) group (**Table 4**, **Figures 2A–D**). The brain metabolic regions negatively correlated with SII or NLR in High_MTV (TLG) group from FIGO stage III were mainly located in the right dlPFC, while some brain regions negatively correlated with NLR were located in the left dlPFC, Occipital_Mid_R, and SupraMarginal_R (**Table 4**). The SII had negative correlations with cerebral glucose metabolism regions in Low_MTV (TLG) group from FIGO stage IV (*P_FWEc_* < 0.05) (**Table 4**, **Figures 2E, F**), but not observed in High_MTV (TLG) group. The brain metabolic regions negatively correlated with SII in Low_MTV (TLG) groups from FIGO stage IV were not only located in the left dlPFC, but also located in Postcentral_R, Parietal_Inf_L(R), Temporal_Sup_L, and Occipital_Mid_L (*P_FWEc_* < 0.05).

**Table 4:** Peripheral inflammatory markers correlated with brain glucose metabolism in high_MTV (TLG) group and low_MTV (TLG) group from FIGO stage III and IV.

Peripheral Inflammatory Markers	Brain Regions (aal)	Voxels	Peripheral Inflammatory Markers	Brain Regions (aal)	Voxels
FIGO stage III					
High_MTV			Low_MTV		
SII	Frontal_Mid_R	1374	SII	No suprathershold clusters	
	Frontal_Sup_R	380			
	Frontal_Inf_Oper_R	317			
	Frontal_Inf_Orb_R	283			
	Frontal_Inf_Tri_R	191			
NLR	Frontal_Mid_R	1788	NLR	No suprathershold clusters	
	Frontal_Inf_Tri_R	519			
	Frontal_Sup_R	412			
	Frontal_Mid_L	335			
	Frontal_Inf_Oper_R	323			
High_TLG			Low_TLG		
SII	Frontal_Mid_R	477	SII	No suprathershold clusters	
	Frontal_Inf_Oper_R	224			
	Frontal_Inf_Tri_R	110			
NLR	Frontal_Mid_R	1224	NLR	No suprathershold clusters	
	Frontal_Inf_Tri_R	327			
	Frontal_Inf_Oper_R	276			
	Frontal_Sup_R	217			
	Occipital_Mid_R	303			
	SupraMarginal_R	237			
FIGO stage IV					
High_MTV			Low_MTV		
SII	No suprathershold clusters		SII	Postcentral_R	861
				Frontal_Mid_L	734
				Parietal_Inf_L	659
				Frontal_Sup_L	457
				Frontal_Mid_R	235
				Frontal_Inf_Orb_L	230
				Parietal_Inf_R	216
				Temporal_Sup_L	199
High_TLG			Low_TLG		
SII	No suprathershold clusters		SII	Frontal_Mid_L	851
				Postcentral_R	761
				Parietal_Inf_L	736
				Frontal_Sup_L	419
				Frontal_Sup_Medial_L	320
				Occipital_Mid_L	270
				Frontal_Inf_Orb_L	239

## Discussion

We use [18F]FDG PET to explore brain [18F]FDG metabolic associations of CC-related peripheral inflammatory markers, and we found that altered brain glucose metabolisms were located in the dlPFC. Further analyses suggest that the relationships between peripheral inflammatory markers and brain metabolism would be stronger for patients with the higher level of MTV or TLG in patients with FIGO stage III, but the relationships between them would be weaker or disappeared in patients with FIGO stage IV.

Beyond cervical cancer, the relationships between tumor metabolic parameters and peripheral inflammatory marker have been also studied in other different cancer types, such as colorectal (8), small cell lung cancer (9, 10), cholangiocarcinoma (11), breast cancer (12) and nasopharyngeal (13). However, their conclusions were not entirely consistent. Our findings showed that primary tumor MTV or TLG had significantly but weakly positive correlation with peripheral inflammatory markers in patients with FIGO stage III or IV, but did not correlate with those in FIGO stage II. Theoretically, the MTV and TLG of primary tumor not only includes the [18F]FDG uptake of tumor tissue itself, but also the [18F]FDG uptake of local inflammation occurs. These significantly positively relationships between primary tumor MTV (TLG) and peripheral inflammatory markers suggest that peripheral inflammatory markers might weakly affect tumor MTV and TLG. Platelets often increase in patients with different solid tumour types, and they are considered to directly contribute to tumour growth (14). Neutrophils, as a marker for the response to systemic inflammation, play critical roles in the production of endothelial growth factor, resulting in the tumor angiogenesis (15). Lymphocytes can suppress cancer cell growth by secreting cytokines, such as IFN-γ or TNF-α (16). The decreased lymphocyte counts in cancer patients may indicated the weakening of immunological surveillance and defense (16, 17). Monocytes also play key roles in the regulation of caner growth and metastasis (18). The counts of monocytes have been confirmed to correlated with survival in patients with locally advanced CC (19). The inflammatory responses contribute to the development and progression of cancer (20, 21). Tavares-Murta et al. (22). demonstrated that the inflammatory markers were significantly higher in the patients with advanced CC. Their results are similar to our findings. The peripheral inflammatory markers were significantly higher in FIGO stage III or IV than those in FIGO stage II, but no significant difference was found between FIGO stage III and FIGO stage IV in the present study. The peripheral inflammatory markers were considered as predictors for the invasiveness of cervical cancer. The sensitivity and specificity of pretreatment PLR and NLR were found to over the MLR for the FIGO stage I-II and III-IV in CC patients. Therefore, the peripheral inflammatory markers might not only reflect the local inflammation, but also reflect the protumoral or antitumoral immune status in CC patients.

The previous neuroimaging studies have identified that the limbic, midbrain, brainstem, the basal ganglia, the insula, ACC, PFC may participate in mediating a series of psychological behavior related to peripheral inflammation (23). Peripheral inflammation is considered to relate with transient and adaptive behaviors included anxiety, altered threat coping strategies, fatigue, hyperalgesia, and social & physical withdrawal (24). These above behaviors are considered an adaptive mechanism to deal with diseases, enhance convalescence, and prevent potential hazards (25). Pomykala et al. (26). found that the medial prefrontal and anterior temporal glucose metabolism was positively associated with pro-inflammatory cytokines in chemotherapy patients with breast cancer, but this relationship was not found in patients without chemotherapy. However, in the present study, the most consistent and negative associations between brain metabolism and peripheral inflammatory markers in CC patients without treatment from FIGO stage III or IV were mainly located in the dlPFC, but no relationship was found between them in FIGO stage II. The relationships between brain regional glucose metabolism and peripheral inflammatory markers may not only might be relevant in the setting of different disorders (cancer, acute and chronic inflammation, and et al.) and treatment methods, but also relevant in the disease severity.

The stronger relationship of hypometabolism in dlPFC with higher peripheral inflammatory markers for CC patients in High_MTV(TLG) group of FIGO stage III would reflect that the relationship in FIGO stage III become stronger as the disease progresses. Furthermore, the brain metabolic regions negatively correlated with SII in Low_MTV(TLG) group of FIGO stage IV were not only located in dlPFC, but also in Postcentral_R, Parietal_Inf_L(R), Temporal_Sup_L, or Occipital_Mid_L, while there was no correlation of the glucose metabolism in dlPFC or any other cerebral regions with higher peripheral inflammatory markers in High_MTV (TLG) group of FIGO stage IV. These findings indicate that although more brain regions of CC patients correlated with peripheral inflammatory markers in FIGO stage IV was found, the relationship of dlPFC with peripheral inflammatory markers in FIGO stage IV weakened or even disappeared with the deterioration of disease. The MTV or TLG of primary tumor didn’t fully reflect the entire disease status of the CC patients in FIGO stage IV due to the more and distant metastasis in FIGO stage IV related to FIGO stage III. However, this cannot sufficiently explain the weakening or even disappearance of the relationship between SII and brain glucose metabolism in FIGO stage IV. Some prior studies reported that brain may regulate tumor growth of peripheral tumors through neuroendocrine-immune system (27), which might be believed to reflect attempts to restore homeostasis (1). From local metastasis in FIGO stage III to distant or organ metastasis in FIGO stage IV, which might imply the further dysregulation or even collapse of internal homeostasis in FIGO stage IV. Hence, the weakening or even disappearance of relationship between SII and brain glucose metabolism might be due to the further dysregulation or collapse of homeostasis in CC patients of FIGO stage IV to some extent. Together, our findings suggest that higher peripheral inflammatory markers may be associated with hypometabolism in the dlPFC of CC patients in the FIGO stage III or IV, which might vary depending on the disease severity.

Interestingly, the high_MTV (TLG) group in FIGO stage III showed hypometabolic regions correlated with higher peripheral inflammatory markers mainly located in right dlPFC, but in the whole group in FIGO stage III showed hypometabolism regions mainly located in bilateral dlPFC. In addition, the low_TLG (MTV) group in FIGO stage IV showed hypometabolic brain regions mainly in left dlPFC, but the whole group in FIGO stage IV showed hypometabolic brain regions mainly located in right dlPFC. These results suggested that during the progression of the disease, the glucose metabolism in the left dlPFC might decrease earlier than those in the right dlPFC of CC patients in FIGO stage III or IV. And a prior study has demonstrated that the left prefrontal region in patients may be vulnerable to the effect of disease severity (28). As we known, the dlPFC plays an important role in working memory, planning, goal-driven attention, problem-solving, and task switching (29). Prior studies reported that the role of left dlPFC, which playing for cognitive processing, is different from right dlPFC playing for attention and decision making (30). Hence, the CC patients in FIGO stage III or IV might firstly experience cognitive impairment, followed by the decline in attention and decision making. However, the potential mechanisms need further research.

There are some shortcoming in the present study. Due to retrospective study, this study lack of education level, age at menarche and parity. Additionally, lacking of the evaluation of neuropsychiatric scale, the neural association of primary tumor burden and peripheral inflammatory markers in CC patients cannot be fully revealed and need further and more research. Despite above shortcomings, the fact that inflammatory markers significantly correlated with regional brain metabolism and those relationships are stronger in FIGO stage III and weaken or disappear in FIGO stage IV for patients with the higher level of tumor burden is quite reliable.

In conclusion, the glucose metabolism in the dlPFC negatively correlated with peripheral inflammatory markers in patients with FIGO stage III or IV may be relevant in the disease severity and vary depending on the disease severity. Based on our findings, more attention is needed for CC patients who have increased peripheral inflammatory markers, MTV, and TLG in different FIGO stage. Further and more researches are needed to confirm our findings.

## Data Availability

The original contributions presented in the study are included in the article/**Supplementary Material**. Further inquiries can be directed to the corresponding author.
