# Supplementary material for: Brian [18F]FDG PET associations of cervical cancer-related peripheral inflammatory markers
Source: Front Oncol. 2025 Jun 26;15:1598911. doi: 10.3389/fonc.2025.1598911 (PMC12260926; doi:10.3389/fonc.2025.1598911)
Supplement: Supplementary file 1 [file Table1.docx]

**Supplementary Table 1** FIGO stage correlated with peripheral inflammatory markers and PET metabolic parameters of primary lesion in CC patients

| **Peripheral Inflammatory Markers** | **FIGO Stage II** | **FIGO Stage III** | **FIGO Stage IV** | **H** | ***P*** |
| --- | --- | --- | --- | --- | --- |
| **SII** | 471.70 (189.95-788.10) | 706.60(135.60-5295.48)a | 744.00(90.04-3695.41)a | 14.333 | 0.001 |
| **PLR** | 117.09 (62.90-232.50) | 167.71(61.72-394.62)a | 171.43(73.79-438.18)a | 12.952 | 0.002 |
| **NLR** | 2.01 (1.25-2.90) | 2.61(0.84-11.61)a | 3.17(0.65-16.72)a | 17.321 | 0.000 |
| **MLR** | 0.20 (0.10-0.30) | 0.26(0.10-0.90)a | 0.28(0.08-0.73)a | 16.544 | 0.000 |
| **MTV** | 13.28(3.26-65.33) | 30.28(2.35-162.30)a | 36.51(2.22-225.15)a | 14.314 | 0.001 |
| **TLG** | 111.07(12.49-442.02) | 287.97(14.44-2183.11)a | 341.34(9.41-2978.09)a | 13.750 | 0.001 |

a: compared with patients with FIGO stage II, *P*<0.05; b:compared with FIGO stage III, *P*<0.05.

**Supplementary Table 2** Characteristics between the Low_MTV(TLG) group and High_MTV(TLG) group in different FIGO stage

| **Characteristics** | **FIGO stage II (16)** | | | **FIGO stage III (160)** | | | **FIGO stage IV (91)** | | |
| --- | --- | --- | --- | --- | --- | --- | --- | --- | --- |
|  | **Low_MTV group (<13.28，8)** | **High_MTV group (≥13.28，8)** | ***P*** | **Low_MTV group (<30.28，80)** | **High_MTV group (≥30.28，80)** | ***P*** | **Low_MTV group (<36.51，45)** | **High_MTV group (≥36.51，46)** | ***P*** |
| **Age (y, median, range)** | 59 (43-68) | 58.5 (37-73) | 0.944 | 52 (28-75) | 53 (26-72) | 0.974 | 53 (36-76) | 52.5 (30-71) | 0.633 |
| **BMI (median, range)** | 23.37 (21.61-28.13) | 22.09 (16.20-28.13) | 0.398 | 23.50 (16.80-32.02) | 23.18 (16.65-45.17) | 0.891 | 23.44 (16.89-30.02) | 24.24 (16.8-29.74) | 0.971 |
| **[18F]FDG(mCi) (median, range)** | 10.03 (6.46-11.74) | 9.96 (7.08-12.32) | 0.941 | 10.17 (6.39-12.86) | 9.76 (5.98-13.17) | 0.485 | 9.92 (6.76-12.32) | 10.41 (7.14-13.13) | 0.125 |
| **SII (median, range)** | 427.48(189.95-711.59) | 471.69(223.61-788.10) | 0.674 | 608.77(135.60-2406.73) | 857.02(185.47-5295.48) | 0.000 | 649.61(90.04-2325.30) | 896.63(212.89-3695.41) | 0.044 |
| **PLR (median, range)** | 113.31(62.90-217.36) | 130.87(76.06-232.48) | 0.651 | 156.16(61.72-375.00) | 189.06(62.01-394.62) | 0.003 | 170.97(84.15-438.18) | 176.68(73.79-420.62) | 0.683 |
| **NLR (median, range)** | 1.88(1.25-2.66) | 2.08(1.35-2.90) | 0.496 | 2.33(0.84-9.86) | 3.12(1.09-11.61) | 0.000 | 3.04(0.65-7.59) | 3.73(0.67-16.72) | 0.162 |
| **MLR (median, range)** | 0.20(0.10-0.24) | 0.19(0.12-0.30) | 0.446 | 0.23(0.10-0.64) | 0.29(0.11-0.90) | 0.000 | 0.28(0.17-0.73) | 0.30(0.08-0.72) | 0.544 |
|  | **Low_TLG group (<111.07，8)** | **High_TLG group (≥111.07，8)** | ***P*** | **Low_TLG group (<287.97，80)** | **High_TLG group (≥287.97，80)** | ***P*** | **Low_TLG group (<341.34，45)** | **High_TLG group (≥341.34，46)** | ***P*** |
| **Age (y, median, range)** | 59 (43-68) | 58.5 (37-73) | 0.944 | 53 (28-76) | 51 (26-72) | 0.477 | 53 (36-76) | 52 (30-71) | 0.322 |
| **BMI (median, range)** | 23.37 (21.61-28.13) | 22.09 (16.20-28.13) | 0.398 | 23.23 (16.8-32.02) | 23.61 (16.65-45.17) | 0.495 | 23.19 (16.89-30.02) | 24.65 (16.80-29.74) | 0.099 |
| **[18F]FDG(mCi) (median, range)** | 10.03 (6.46-11.74) | 9.96 (7.08-12.32) | 0.941 | 10.04 (6.39-12.86) | 9.84 (5.98-13.17) | 0.787 | 9.92 (6.76-12.32) | 10.41 (7.35-13.13) | 0.081 |
| **SII (median, range)** | 427.48(189.95-711.59) | 471.69(223.61-788.10) | 0.674 | 636.43(135.60-2250.00) | 852.39(185.47-5295.48) | 0.000 | 646.76(90.04-2325.30) | 967.59(212.89-3695.41) | 0.014 |
| **PLR (median, range)** | 113.31(62.90-217.36) | 130.87(76.06-232.48) | 0.651 | 156.91(61.72-375.00) | 179.98(62.01-394.62) | 0.005 | 168.21(84.15-438.18) | 181.48(73.79-420.62) | 0.487 |
| **NLR (median, range)** | 1.88(1.25-2.66) | 2.08(1.35-2.90) | 0.496 | 2.35(0.84-7.05) | 3.03(1.09-11.61) | 0.004 | 2.85(0.65-7.59) | 3.77(0.67-16.72) | 0.054 |
| **MLR (median, range)** | 0.20(0.10-0.24) | 0.19(0.12-0.30) | 0.446 | 0.24(0.10-0.79) | 0.28(0.10-0.90) | 0.007 | 0.27(0.17-0.73) | 0.30(0.08-0.72) | 0.389 |
